# Effects of Soybean Meal Substitution in Finishing Pig Diet on Carcass Traits, Meat Quality, and Muscle Antioxidant Capacity

**DOI:** 10.3390/ani15111611

**Published:** 2025-05-30

**Authors:** Shuai Liu, Zhentao He, Xiaolu Wen, Xianliang Zhan, Lei Hou, Dongyan Deng, Kaiguo Gao, Xuefen Yang, Shuting Cao, Zongyong Jiang, Li Wang

**Affiliations:** 1 State Key Laboratory of Swine and Poultry Breeding Industry, Key Laboratory of Animal Nutrition and Feed Science in South China, Ministry of Agriculture and Rural Affairs, Guangdong Laboratory for Lingnan Modern Agriculture, Heyuan Branch, Guangdong Key Laboratory of Animal Breeding and Nutrition, Institute of Animal Science, Guangdong Academy of Agricultural Sciences, Guangzhou 510640, China; 15027016245@163.com (S.L.); kuzma022133@gmail.com (Z.H.); wenxiaolu@gdaas.cn (X.W.); zhanxianliang1996@163.com (X.Z.); rhoulei@126.com (L.H.); 15675858457@163.com (D.D.); gaokaiguo312@126.com (K.G.); yangxuefen@gdaas.cn (X.Y.); jiangzy@gdaas.cn (Z.J.); 2 College of Animal Science, South China Agricultural University, Guangzhou 510642, China

**Keywords:** finishing pig, substitution for soybean meal, meat quality, mixed meal, antioxidant

## Abstract

This study evaluated the effect of mixed meal replacement of soybean meal on growth conditions, carcass traits, and meat quality of finishing pigs by partially and entirely replacing soybean meal with equal proportions of rapeseed, cotton, and sunflower meal. A total of fifty-four pigs with an average initial weight of 97.60 ± 0.30 kg were selected and randomly divided into three groups according to their initial weight, with six pens in each group and three pigs in each pen. The experimental groups were as follows: control group (CON), fed corn–soybean meal type basal diet; corn–soybean mixed meal group (CSM), using equal proportions of rapeseed meal, cotton meal, and sunflower meal (3.52% each) to replace 9.06% of soybean meal in the basal diet; and corn mixed meal group (CMM), using equal proportions of rapeseed meal, cotton meal, and sunflower meal (6.46% each) to replace soybean meal in the basal diet completely. According to the results, the use of mixed meal as a replacement for soybean meal did not have a significant impact (*p* > 0.05) on the average daily weight gain, average daily feed intake, feed-to-weight ratio, body size, carcass traits, and meat quality of finishing pigs. The entire replacement of soybean meal with a mixed meal resulted in a significant increase (*p* < 0.05) in leaf fat weight. The use of mixed meal as a substitute for soybean meal had no significant effect (*p* > 0.05) on the antioxidant capacity and fatty acid composition of the longissimus thoracis in finishing pigs. However, longissimus thoracis muscle fiber diameter was reduced in the mixed meal partially replaced soybean meal group compared to the mixed meal completely replaced soybean meal group (*p* < 0.05). In addition, mixed meal replacing soybean meal did not significantly affect (*p* > 0.05) the expression of the longissimus thoracis muscle fiber type genes *MYHC1* and *MYHC2*. Mixed meal replacement of soybean meal did not significantly affect (*p* > 0.05) the expression of *ACACA*, *FASN*, and *PPARG* genes in the longissimus thoracis. This study showed that mixed meal as an alternative to soybean meal in diets did not have significant negative effects on the growth performance and meat quality of finishing pigs. These results can help develop further mixed meals as a functional alternative feed ingredient for soybean meals in pig diets.

## Simple Summary

Soybean meal is the main protein ingredient in livestock and poultry feed. With the fluctuation of soybean meal prices, overdependence on soybean meal can lead to greater cost risk. Mixed meals such as rapeseed meal, cotton meal, and sunflower meal have great potential to replace soybean meal. This study shows that partial and total replacement of soybean meal in diets using equal proportions of rapeseed meal, cotton meal, and sunflower meal was found to have no significant effect on growth, carcass traits, and meat quality of finishing pigs. The results of this study provide a rationale for the substitution of soybean meal by mixed meals, which will help to further develop and utilize mixed meal resources.

## 1. Introduction

Soybean meal is the most important protein raw material in the feed industry and the main source of protein in livestock and poultry production [1]. A byproduct of soybean oil extraction, SBM possesses a crude protein content ranging from 40% to 50% [2]. In addition, the amino acid composition of soybean meal is also very similar to that of livestock and poultry and has a high lysine content [3]. Soybean meal exhibits high biological efficiency, with standardized ileal digestibility of most amino acids approaching 90% in poultry [4] and exceeding 90% for nearly all amino acids in pigs [5]. However, anti-nutritional factors in soybean meal, such as trypsin inhibitors and lectins, hinder nutrient absorption. Furthermore, the environmental impact of soybean meal utilization and its price volatility pose significant risks to the agricultural industry [6,7]. Consequently, reducing reliance on soybean meal and identifying suitable alternative protein sources have become critical research focuses.

For decades, unconventional feed ingredients such as mixed meals (cottonseed meal, rapeseed meal, and sunflower meal), dregs, woody feeds, etc. have been investigated as potential replacements for soybean meal [8,9]. This exploration of alternative protein sources is driven by the need for more sustainable and economically viable pig production systems. Cottonseed meal, a byproduct of cottonseed oil extraction, has a crude protein content ranging from 30% to 50%, depending on the hulling efficiency and oil extraction rate [10]. While the presence of gossypol and other cotton phenols inhibits digestive enzymes, potentially impacting nutrient utilization and growth performance in pigs. Mixed meals (cottonseed meal, rapeseed meal, and sunflower meal) have lower crude protein levels than soybean meal and higher levels of sulfur amino acids, which have the potential to replace soybean meal [11,12]. However, further studies are warranted to evaluate the long-term effects of mixed meal inclusion on pig health and productivity. These findings highlight the potential of oilseed meals as sustainable and cost-effective protein sources in pig diets, warranting further investigation into optimizing their inclusion levels and mitigating potential antinutritional factors to maximize their benefits for pig production.

Carcass traits and meat quality traits are key determinants of the economic value of finishing pigs [13]. Therefore, this study investigated the effects of partially or completely replacing soybean meal with a mixture of rapeseed meal, cottonseed meal, and sunflower meal in the diets of finishing pigs during the later stages of finishing. The objective was to determine whether this dietary substitution adversely affects carcass traits, meat quality, antioxidant capacity, and fat metabolism, and ultimately, to provide a theoretical basis for formulating suitable soybean meal replacement strategies.

## 2. Materials and Methods

### 2.1. Animals and Experimental Diets

All animal treatment protocols in this study were approved by the Animal Care and Use Committee of the Guangdong Academy of Agricultural Sciences and followed the Guide for the Care and Use of Animals for Research and Teaching (Authorization No. GAIASIAS-2022-022).

A total of 54 pigs (Landrace × Yorkshire × Duroc, 27 barrows and 27 gilts) with an average initial weight of 97.60 ± 0.30 kg were selected and randomly divided into 3 treatment groups according to their initial weight. Each treatment will encompass 6 replicates with 3 pigs per pen (9 barrows and 9 gilts). The trial period was 26 days. The groups were as follows: control group (CON), fed corn–soybean meal type basal diet; corn–soybean mixed meal group (CSM), fed corn–soybean mixed meal diet with a ratio of rapeseed meal, cotton meal, and sunflower meal (3.52% each) to replace 9.06% soybean meal in the basal diet; corn mixed meal group (CMM), fed a corn mixed meal diet with a ratio of rapeseed meal, cotton meal, and sunflower meal (6.46% each) to entirely replace soybean meal in the basal diet. Crude protein levels were maintained at 12.5% for all three diets. The trial diets were formulated according to the National Research Council [14] for finishing pigs. The composition of the basal diet is shown in Table 1. The fatty acid composition and profile of the feed are shown in Supplementary Figure S1 and Table S1. The nutrient components of rapeseed meals, cottonseed meals, and sunflower seed meals are shown in Supplementary Table S2. Diets were analyzed for nitrogen by the method 984.13 of AOAC (1990) [15] using a Kjeltec 8400 apparatus (FOSS Inc., Eden Prairie, MN, USA), and crude protein was calculated as nitrogen × 6.25. The gross energy was measured by an oxygen bomb calorimeter Parr 6400 (Parr Instrument Company, Moline, IL, USA). Ether extract was determined using method 920.39 of AOAC (1995) [16] by Automatic Fat Analyser XT15i (Ankom Technology Corporation, Fairport, NY, USA). Glycinin, β-conglycinin, trypsin inhibitor, and agglutinin of the diets were determined by using an enzyme-linked immunoassay kit (Tianjin Longke-truthers Biotechnology Co., Ltd., Tianjin, China). The measurements were performed with reference to the kit instructions. All the kits were purchased from Tianjin Longke-truthers Biotechnology Co., Ltd (Tianjin, China). All 18 pens were identical, with the same covered area (3 m^2^/pig), and were equipped with similar troughs for feed concentrates and water. Pigs were provided ad libitum access to water and feed during the entire experimental trial.

### 2.2. Sample Collection and Measurements

At the end of the experiment, the pigs were weighed after 12 h of fasting. One pig per pen was randomly selected to be slaughtered. At the end of the trial, we slaughtered 6 pigs per group for sampling and for later analysis. Body height (vertical distance from the highest point of the scapula to the ground), body length (length from the occipital ridge to the root of the tail), chest circumference (length of the posterior angle of the scapula around the chest perpendicular to the body axis), and abdomen circumference (thickest part of the abdomen) were measured using a tape measure. Each pig had 10 mL of venous blood taken at the ear margin, kept at room temperature for 40 min, then centrifuged (4 °C, 2000 rpm) to extract the supernatant, frozen in liquid nitrogen, and transferred to the −80 °C refrigerator for storage. All experimental pigs were stunned, bled, debrided, gutted, and cut from the midline according to commercial procedures. All slaughtering procedures and sampling procedures were carried out in the slaughterhouse (about 100 m away from the pig house), in the cold room inside the slaughterhouse, and in the laboratory inside the slaughterhouse as required by the project. The hot carcass, leaf fat, heart, liver, spleen, lung, and kidney were weighed and recorded. Organ index (%) = (organ weight/live weight) × 100. Carcass yield (%) = hot carcass weight/live weight × 100. The backfat thickness was measured using vernier calipers on the left side of the carcass at the first rib, the tenth rib, the last rib, and the lumbosacral junction, and the average backfat thickness was calculated. Using a pencil on transparent sulfate paper, the left carcass was drawn in the loin–eye profile at the thoracolumbar junction and the profile area using a digital planimeter (KP90N, Koizumi, Osaka, Japan). After that, longissimus thoracis et lumborum from the fourth rib to the dorsal region near the rump were excised from the left side of the carcass for meat analysis (within 20 min after death). The longissimus thoracis (LT) of the back (1 cm × 1 cm × 1 cm) was fixed in 4% paraformaldehyde for H&E staining, and another piece was placed in liquid nitrogen and transferred to −80 °C for storage.

### 2.3. Meat Quality Determination

Flesh color was measured with reference to the method of Biffin et al. [17]. The color of the LT was measured at 45 min, 24 h, and 48 h (blooming time for 20 min) after slaughter by a D-65 light source colorimeter (CR-410, Minolta, Chiyoda, Japan), and L* (brightness), a* (redness), and b* (yellowness) values were recorded. Before using the colorimeter, calibrate it with a white calibration plate with a 50 mm measuring aperture. Measurements are made without covering the film. The pH value of the LT at 45 min, 24 h, and 48 h after slaughter was measured using a pH meter (Testo-205, Desto, Lenzkirch, Germany). Calibrate the pH meter at room temperature (temperature compensation in automatic mode) using pH 4.01 and 7.00 buffers (Desto, Lenzkirch, Germany). All pigs were slaughtered on the same day. The muscles were stored in a cold room at 4 °C for 48 h. Samples of 5 cm × 3 cm × 2 cm long LT strips were weighed and suspended in aerated polyethylene film bags with fishhooks for 24 h and 48 h at 2–4 °C. The fishhooks were removed, dried on filter paper, and reweighed. Drip loss (%) = (weight before suspension − weight after suspension)/weight before suspension × 100. LT marbling was assessed by 10 different assessors with reference to a standard scale for pork marbling (NPPC, 1991) [18]. Immediately after slaughter, samples of muscle were taken and stored at 2–4 °C for 24 h. After 24 h, the samples were removed and placed in a plastic bag and heated in a water bath at 80 °C until the internal temperature reached 70 °C, then immediately removed and brought to room temperature. Ten cylindrical meat samples were taken parallel to the direction of the muscle fibers using a standard sampler (1.27 cm in diameter), and muscle shear force was measured using a digital tenderness meter (C-LM3B, Northeast Agricultural University College of Engineering, Harbin, China).

The fatty acid profile of LT was determined uing gas chromatography [19]. In brief, 10 g (accurate to 0.1 mg) of the sample was hydrolyzed by adding 2.0 mL of undecylenic triglyceride internal standard solution and 100 mg of pyrogallic gallic acid, 2 mL of 95% ethanol, 4 mL of water, and 10 mL of hydrochloric acid. The hydrolyzed product was extracted using ether and evaporated to dryness. The residue after evaporation was saponified by adding sodium hydroxide methanol and then methyl esterified by adding methanolic boron trifluoride. Finally, n-heptane and saturated aqueous sodium chloride were added and mixed. After standing and layering, the supernatant strip was taken for determination. A single standard solution of fatty acid methyl esters and a mixed standard solution of fatty acid methyl esters were injected into the gas chromatograph, and the chromatographic peaks were characterized. The fatty acid standard solution and the sample solution were injected into the gas chromatograph, and the peak areas were quantified by the chromatographic peaks.

Determination of inosinic acid content in muscle using high-performance liquid chromatography. Two grams (accurate to 0.0001 g) of fresh muscle were weighed and cut into 50 mL centrifuge tubes; 20 mL of pre-cooled 5% perchloric acid solution was added accurately, homogenized for 1 min at 8000 rpm using a high-speed tissue homogenizer, then transferred to 50 mL volumetric flasks and fixed with 5% perchloric acid. The solution was centrifuged at 4000 rpm for 3 min, and 10 mL of the supernatant was accurately pipetted. The pH was adjusted to 6.5 with 0.5 mol/L sodium hydroxide solution, and the solution was transferred to a 50 mL volumetric flask and fixed with water. It was filtered with a 0.45 μm filter membrane and then determined by liquid chromatography.

### 2.4. Antioxidant Capacity Evaluation

All the kits used for these measurements were procured from the Nanjing Jiancheng Institute of Biological Engineering (Nanjing, China). The Total Antioxidant Capacity (T-AOC) was assessed using the ABTS method (Product No. A015-2-1, Nanjing Jiancheng Corporation, Nanjing, China), while the malondialdehyde (MDA) content was gauged using the TBA method (Product No. A003-2, Nanjing Jiancheng Corporation, Nanjing, China). The catalase (CAT) activity was evaluated using the ammonium molybdate method (Product No. A007-1-1, Nanjing Jiancheng Corporation, Nanjing, China), and the total superoxide dismutase (T-SOD) activity was quantified using the hydroxylamine method (Product No. A001-1, Nanjing Jiancheng Corporation, Nanjing, China). The samples from each LT were homogenized and analyzed as per the guidelines provided with the kits. Briefly, the samples were homogenized with the sample dilution at a ratio of 4:10. The homogenate was centrifuged at 4500 rpm for 10 min at 4 °C. The supernatant was taken for the enzymatic assay. The protein concentrations of the sample were determined with a Bradford Protein Concentration Assay Kit (Beyotime, Shanghai, China), and the results were expressed per mg protein.

### 2.5. Histochemistry Staining

The classical H&E staining method was used to determine the muscle fiber characteristics of the LT. The LT was excised vertically from the left carcass at the junction of the thoracolumbar segment. Tissue sections were obtained after fixation with a 4% formaldehyde solution, tissue repair and dehydration, paraffin embedding, sectioning, dewaxing, H&E staining, dehydration, and neutral gel sealing. Images were observed and collected using a Nikon Eclipse E100-DS-U3 microscope (Nikon, Tokyo, Japan) imaging system, and muscle fibers were analyzed using Case Viewer software (1.4.0.50094, 3DHISTRCH Ltd., Budapest, Hungary). Five fields of view were randomly selected from a sample, and 10 fibers were randomly selected in each field of view. The muscle fiber diameter (μm) was the average length of the long and short axes of the 10 fibers measured in each pig. Also, the myofiber density (mm^2^) of a sample was calculated from the area of the 5 random fields and the number of myofibers in the corresponding area. Myofiber density = number of myofibers in the myofield/selected area of the myofield.

### 2.6. RNA Extraction, cDNA Synthesis, and Real-Time Quantitative PCR (RT-qPCR)

RNA extraction was performed using TRIzol reagent (Invitrogen, Carlsbad, CA, USA) following the manufacturer’s protocol from samples of LT. Complementary DNAs (cDNAs) were synthesized from 1 μg of total RNA using a reverse transcription kit (TaKaRa, Tokyo, Japan). The resulting cDNA was diluted (1:10, *v*/*v*) and subjected to real-time quantitative polymerase chain reaction (qPCR) amplification using specific primers for porcine messenger RNA (mRNA) sequences (Supplementary Table S1) and SYBR green I (TaKaRa). The qPCR conditions included an initial denaturation at 95 °C for 180 s, followed by 40 cycles at 95 °C for 15 s and 58 °C for 30 s, with a final elongation at 72 °C for 30 s. The expression of the target genes relative to the β-actin was evaluated by the 2–ΔΔCT method: ΔCT = CT (target gene) − CT (β-actin) and ΔΔCT = ΔCT (CSM/CMM pigs) − ΔCT (CON pigs).

### 2.7. Statistical Analysis

All data were subjected to one-way analysis of variance (one-way ANOVA) using the IBM SPSS Statistics V25.0 software package (IBM Corporation, Armonk, NY, USA). All data were tested for normal distribution prior to ANOVA. Homogeneity of variance was tested using Levene’s test. ANOVA followed by Tukey’s test. Significance was set at *p* < 0.05.

## 3. Results

### 3.1. Growth Performance, Body Size, and Organ Indexes

Replacing soybean meal with the mixed meal had no significant effect on average daily feed intake, average daily gain, and ratio of feed to gain [20].

As shown in Table 2, replacing soybean meal with mixed meal had no significant effect (*p* > 0.05) on the body weight, length, height, or chest circumference of finishing pigs. While abdominal circumference tended to be greater in the cottonseed meal (CSM) group compared to the control (CON) group (0.1 > *p* > 0.05), this difference was not statistically significant. Similarly, partial or complete replacement of soybean meal with the mixed meal did not significantly affect (*p* > 0.05) the organ indices of the heart, liver, lungs, spleen, or kidneys in finishing pigs.

### 3.2. Carcass Traits

Table 3 presents the effects of mixed meal replacement on carcass traits, revealing no significant differences (*p* > 0.05) in carcass straight length, carcass oblique length, carcass weight, or carcass yield. However, complete replacement of soybean meal with the mixed meal resulted in a significant increase (*p* < 0.05) in the leaf fat weight. Partial replacement of soybean meal by mixed meal tended to increase (0.1 > *p* > 0.05) the average backfat thickness of finishing pigs compared to the control group. The replacement of soybean meal with mixed meal had no significant effect (*p* > 0.05) on the loin–eye area of finishing pigs.

### 3.3. Meat Quality

The effect of mixed meal replacement of soybean meal on the meat quality of finishing pigs is shown in Table 4. The analysis revealed no significant differences (*p* > 0.05) in pH values of finishing pig muscle in the CSM and CMM groups at 45 min, 24 h, and 48 h compared to the CON group. Similarly, the substitution with a mixed meal diet in place of soybean meal also had no significant effect (*p* > 0.05) on flesh color, drip loss, muscle shear force, and marbling.

### 3.4. Muscle Fatty Acid Composition

The effect of mixed meal replacement of soybean meal has no effect (*p* > 0.05) on the longissimus lumborum fatty acid content of finishing pigs is shown in Table 5.

### 3.5. Muscle Antioxidant Capacity

In Table 6, replacing soybean meal with the mixed meal did not significantly affect (*p* > 0.05) MDA content, T-AOC content, CAT viability, and T-SOD viability in the LT of finishing pigs.

### 3.6. Muscle Fiber Density and Diameter

**Figure 1:**
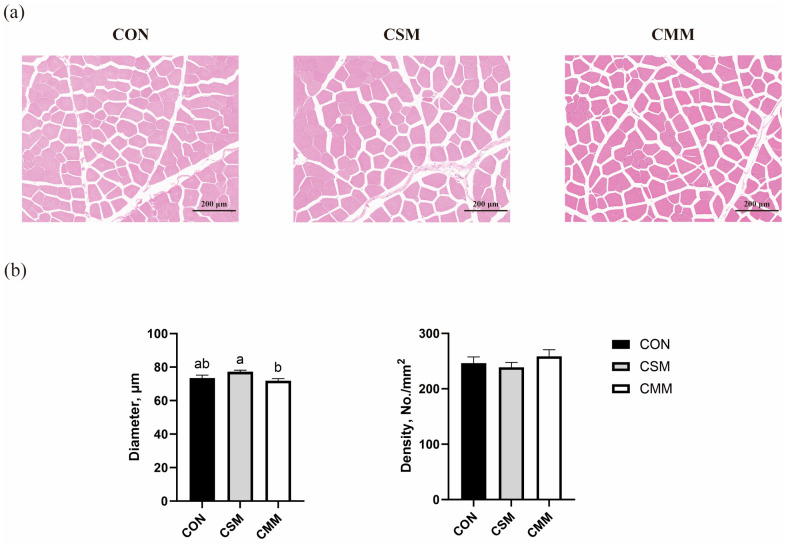
Effect of mixed meal replacement of soybean meal on the longissimus thoracis fiber density and diameter of finishing pigs. (**a**) H&E staining of longissimus thoracis magnification × 12. (**b**) myofiber density and diameter. CON, the control group was fed a corn–soybean meal basal diet; CSM, the corn–soybean mixed meal group was fed with mixed meal to partially replace soybean meal in the basal diet; CMM, the corn mixed meal group was fed with mixed meal as a complete replacement of soybean meal in the basal diet. Values are means ± SEM, *n* = 6. Labeled means in a row with different letters differ, *p* < 0.05.

Figure 1a shows H&E staining pictures of the LT from each experimental group. As shown in Figure 1b, muscle fiber diameter was significantly decreased (*p* < 0.05) in the CMM group compared to the CSM group. However, the replacement of soybean meal with mixed meal has no significant effect (*p* > 0.05) on muscle fiber density in finishing pigs.

### 3.7. The mRNA Expression of Muscle Fiber Type and Lipid Deposition-Related Genes in the LT Muscles

**Figure 2:**
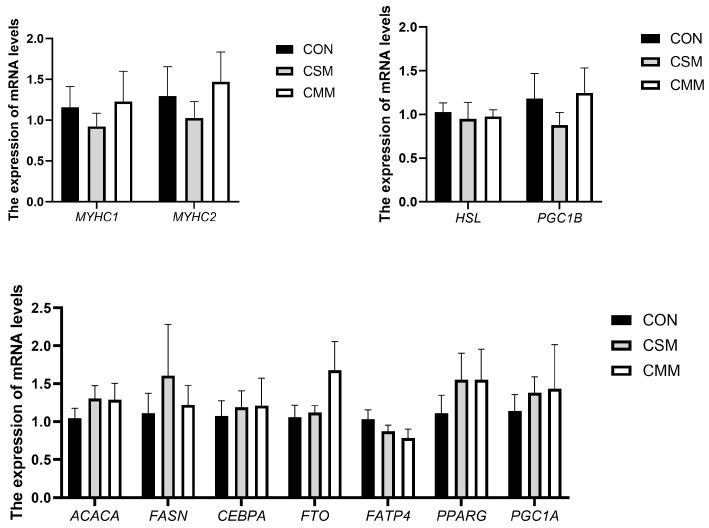
Effect of mixed meal replacement of soybean meal on the type of muscle fiber, fat metabolism, and intramuscular fat deposition genes in the longissimus thoracis of finishing pigs. *MYHC1*, myosin heavy chain 1; *MYHC2*, myosin heavy chain 2; *ACACA*, fatty acid synthesis-related gene acetyl-CoA carboxylase alpha; *FASN*, fatty acid synthase; *CEBPA*, CCAAT enhancer binding protein alpha; *FTO*, FTO alpha-ketoglutarate-dependent dioxygenase; *FATP4*, solute carrier family 27 member 4; *PPARG*, peroxisome proliferator activated receptor gamma; *PGC1A*, PPARG coactivator 1 alpha; *HSL*, hormone-sensitive lipase; *PGC1B*, PPARG coactivator 1 beta. CON, the control group was fed a corn–soybean meal basal diet; CSM, the corn–soybean mixed meal group was fed with mixed meal to partially replace soybean meal in the basal diet; CMM, the corn mixed meal group was fed with mixed meal as a complete replacement of soybean meal in the basal diet. Values are means ± SEM, *n* = 6.

The expression of genes related to muscle fiber types and lipid deposition in the LT is shown in Figure 2. Compared with the CON group, the partial and complete replacement of soybean meal with mixed meal had no significant effect (*p* > 0.05) on the expression of myosin heavy chain 1 (*MYHC1*) and myosin heavy chain 2 (*MYHC2*) genes in the LT. Compared with the control group, the expression of fatty acid synthesis-related genes acetyl-CoA carboxylase alpha (*ACACA*), fatty acid synthase (*FASN*), CCAAT enhancer binding protein alpha (*CEBPA*), FTO alpha-ketoglutarate-dependent dioxygenase (*FTO*), solute carrier family 27 member 4 (*FATP4*), peroxisome proliferator activated receptor gamma (*PPARG*), and PPARG coactivator 1 alpha (*PGC1A*) was not significantly affected (*p* > 0.05) in the LT in the partial and complete replacement of soybean meal with mixed meal group. Compared with the control group, the partial and complete replacement of soybean meal with mixed meal had no significant effect (*p* > 0.05) on the expression of hormone-sensitive lipase (*HSL*) and PPARG coactivator 1 beta (*PGC1B*) genes in the LT.

## 4. Discussion

The feed utilization of mixed meal as a protein resource was feasible. Studies suggest that dietary inclusion of 8–15% cottonseed meal is safe for pigs when free gossypol levels are maintained below 100 mg/kg [11,21]. Rapeseed meal is a by-product of rapeseed oil extraction; the crude protein content is generally 35–40% [22]. Double-low rapeseed meal varieties (low erucic acid and low glucosinolate content) can have crude protein levels as high as 43.6% [23,24]. Rapeseed meal, while slightly lower in lysine than soybean meal, is richer in sulfur-containing amino acids, making it a viable alternative protein source for pigs. Sunflower meal is a by-product of sunflower oil extraction and has a crude protein content of around 30% [25]. Unlike cottonseed meal and rapeseed meal, sunflower meal is generally considered free of significant anti-nutritional factors, simplifying its inclusion in pig feed formulations. While lower in lysine than soybean meal, sunflower meal provides higher levels of sulfur-containing amino acids [12,26]. Research indicates that incorporating up to 16% sunflower meal in finishing pig diets does not negatively impact performance [27]. Consistent with our previous findings of no significant growth performance effects when replacing soybean meal with mixed meals in finishing pigs [28,29], the present study also observed no adverse effects [20]. This is consistent with the established strong correlation between body size and body weight in pigs [30,31]. While replacing soybean meal with cottonseed meal, sunflower meal, and rapeseed meal can reduce dietary lysine levels, lysine was balanced across experimental diets to ensure adequate availability. This is crucial because lysine deficiency can impair protein digestion and absorption, leading to compromised growth [32,33]. However, as noted by Cortamira et al. [34], supplementing lysine and oil may be necessary when utilizing mixed meals. Therefore, careful consideration of the balance among mixed meal components, amino acid levels, and oil supplementation is essential to mitigate potential risks associated with dietary modifications.

Previous studies have found that the use of rapeseed meal in the diet of finishing pigs at a level not higher than 30% does not affect the performance of finishing pigs [35,36]. In a related study, replacing 6% of soybean meal with 3% of rapeseed meal and 3% of cotton meal was found to have no adverse effect on the performance of growing pigs [37]. Furthermore, the addition of 16% sunflower meal in the diets of finishing pigs was found to have no significant adverse effect on carcass traits [27], and even inclusion levels of 21% sunflower meal did not significantly alter backfat depth, carcass weight, or carcass yield in growing and finishing pigs [38]. This aligns with our observations, wherein the substitution of soybean meal with mixed meals did not modify the carcass traits of finishing pigs, corroborating the body size measurements reported previously. However, we were surprised that the entire substitution of soybean meal for a mixed meal resulted in significantly higher weights of plate oil. Dietary fat can be directly converted to body fat, whereas the synthesis of glycerol and fatty acids via the glucose pathway is less efficient [39,40]. Rapeseed meals, sunflower meals, and cotton meals all contain high levels of cellulose and lignin compared to soybean meals. Therefore, we added a certain amount of soybean oil to the mixed meal group for the energy balance of the diet. With the complete replacement of soybean meal, more soybean oil was added, which may have contributed to the heavier leaf fat in the CMM group. However, it was also found that the use of oil in the diet did not cause significant changes in body fat [41]. Therefore, the reasons for the increase in leaf fat weight in the present study need to be further investigated. The thickness of muscle fibers affects the tenderness of meat, and our study found that the diameter of muscle fibers of the longissimus thoracis was significantly higher in the CSM group than in the CMM group. Although the tenderness of the longissimus thoracis of the CSM group was lower than that of the CMM group, this difference was not significant. There was no significant change in the expression of key genes affecting muscle fibers. These results suggest that strategically replacing soybean meal with mixed meals can be a viable strategy for diversifying protein sources in pig nutrition without compromising most key carcass traits. This approach may offer opportunities to enhance sustainability and reduce reliance on traditional soy-based feeds.

Meat quality directly affects the food and commodity value of pork. Our results indicate that the use of mixed meal in place of soybean meal during the finishing period does not alter meat quality at the time of slaughter. A study by Skugor et al. [42] found no significant change in meat quality in pigs fed diets containing 20% rapeseed meal compared to those fed soybean meal-based diets. Furthermore, a mixed diet of 18% rapeseed meal and 16% broad beans was used to replace a basal diet of 14% soybean meal and 6% rapeseed meal, and results showed improved meat color in the replacement group [43]. It has been suggested in the literature that excessive use of sunflower meal may lead to increased linoleic acid in backfat [44]. As yet, there have been no studies on the effects of using cottonseed meal on pork quality. This may be related to the uncertainty of the content of cotton phenols in cottonseed meal. Overall, the use of rapeseed meal, cottonseed meal, and sunflower meal in equal proportions to replace soybean meal did not present a risk of reducing meat quality in our study.

Intramuscular fat, also known as marbling, differs from subcutaneous and visceral fat in that it is found in the interstitial spaces between the muscle fibers and has a more important influence on the flavor, aroma, and tenderness of the meat [45]. Zmudzińska et al. [46] found that using 6% rapeseed meal and legume seeds did not alter the relative content of moisture, protein, or fat in the back muscles of growing pigs. While Grabež et al. [43] observed a decrease in the relative content of protein and moisture in muscle when an 18% rapeseed meal and 16.1% faba bean diet replaced a 14.2% soybean meal and 6% rapeseed meal diet, this difference was not statistically significant. Similarly, in the present study, although muscle total fatty acid content tended to be lower in the mixed meal group, these differences were not statistically significant compared to the control group. The difference is that Grabež’s study found a significant reduction in muscle fatty acid content after replacing soybean meal, whereas in our study there was also a reduction in muscle fatty acid content after replacing soybean meal, but there was no statistical difference [43]. Despite the different composition of diets in this study, there was no significant change in feed intake and composition in muscle, which may be related to the energy balance in the diets and the maturation and plasmolysis during diet processing. Pig feeding behavior, lipogenesis, and protein deposition are closely regulated by nutrient-sensing pathways [47]. The energy supply in the diet remains unchanged, while the intestinal fatty acid sensors are stimulated unchanged, and consequently the genes involved in adipogenesis do not change; protein deposition is similar. The key lipogenic and muscle fiber genes were also not significantly altered in this study, probably as a result of the dynamic regulation of the nutrient-sensing pathway. Inosinic acid is a product of cell metabolism, which can directly determine the fresh flavor of muscle and is an important index used to evaluate the fresh flavor of meat [48]. Although it has been shown that replacing soybean meal in feed changes the metabolomics and flavor of meat [48], the results of this study showed no significant changes in inosinic acid. Therefore, further studies are needed regarding the changes in inosinic acid content and flavor of meat altered by substitution of soybean meal. Overall, replacing soybean meal with the mixed meal did not adversely affect the nutrient composition of pork in the present study.

In the food industry, antioxidants are often used to treat and process meat and meat products, and fresh meat is very susceptible to spoilage if not treated [49]. This process is related to the oxidation of nutrients, specifically the oxidation of fatty acids in food, protein deterioration, etc. In turn, the oxidation of meat can lead to a loss of color and flavor [50,51]. In addition to artificially added antioxidants and antioxidant measures, the strength of the pork’s antioxidant capacity also determines the meat’s preservation period [52,53]. Although unconventional feed ingredients such as mixed meals, dregs, and woody feeds have the potential to enhance the antioxidant capacity of animals, no studies have been reported on the use of mixed meals to increase the antioxidant capacity of pigs [54,55,56]. The results of our study showed that the replacement of soybean meal with mixed meal did not adversely affect muscle antioxidant capacity. The diameter and density of muscle fibers usually show a high correlation with meat quality [57]. Our results showed that the replacement of soybean meal with a mixed meal did not affect muscle fiber diameter and density, but surprisingly there was a significant difference in muscle fiber diameter between the partial and entire replacement groups. The reason behind this discrepancy remains unclear at this moment, necessitating further investigation.

It was shown that muscle fiber diameter was negatively correlated with the expression of the *MYHC1* gene, and that *MYHC* expression could be a potential factor in assessing meat tenderness [58]. In this study, the use of mixed meal instead of soybean meal did not affect the expression of *MYHC*. Adipogenic and lipogenic genes, such as *FASN* and *PPARG*, have been shown to play a crucial role in fatty acid synthesis, triglyceride synthesis, and lipid storage in genetically modified animal models [59,60]. The supplementation of rapeseed meal has been shown to enhance the expression of genes related to lipolysis and fatty acid oxidation in the muscles of growing–finishing pigs [42]. However, in our study, the partial and complete replacement of soybean meal with mixed meal had no significant effect on the expression of genes related to both fat synthesis and metabolism. Compared to the study by Skugor et al. [42] (body weight 29–64 kg), the pigs in our experiment weighed 100–130 kg. However, the specific impacts of substituting soybean meal with alternative meals, including rapeseed meal, cottonseed meal, and sunflower seed meal, on fat metabolism and intramuscular fat deposition remain underexplored. Consequently, this area warrants further investigation to elucidate the potential implications of such dietary modifications on the expression of genes integral to lipid metabolism in pigs.

## 5. Conclusions

In this study, partial and total replacement of soybean meal in diets using equal proportions of rapeseed meal, cotton meal, and sunflower meal was found to have no significant effect on growth, carcass traits, and meat quality of finishing pigs. In conclusion, this study shows that the rational use of mixed meal to replace soybean meal is an effective way to alleviate the raw material crisis and reduce costs without changing the health status of finishing pigs.

## Figures and Tables

**Table 1:** Composition and nutrient level of the experimental diets (as-fed basis).

Ingredients, %	Treatments ^1^		
	CON	CSM	CMM
Corn	56.05	52.94	50.15
Cassava	25.00	25.00	25.00
Soybean meal	16.46	7.40	
Double-low rapeseed meal		3.52	6.46
Cottonseed meal		3.52	6.46
Sunflower meal		3.52	6.46
Soybean oil		1.50	2.75
Calcium carbonate	0.60	0.60	0.58
Calcium phosphate	0.09	0.04	0.03
NaCl	0.40	0.40	0.40
L-lysine hydrochloride	0.08	0.21	0.33
L-threonine	0.01	0.04	0.06
L-tryptophan			0.01
Choline chloride	0.10	0.10	0.10
Premix ^2^	1.21	1.21	1.21
total	100.00	100.00	100.00
Calculated composition 5 ^3^			
Metabolic energy, MJ/kg	13.86	13.80	13.75
Crude protein	12.50	12.50	12.50
Ca	0.49	0.49	0.49
STTD P	0.22	0.22	0.22
SID Lys	0.61	0.61	0.61
SID Met	0.18	0.19	0.20
SID Met + Cys	0.38	0.39	0.40
SID Thr	0.40	0.40	0.40
SID Trp	0.13	0.11	0.11
SID Val	0.48	0.47	0.46
SID Ile	0.45	0.40	0.35
Analyzed value			
Gross energy, MJ/kg	15.65	16.08	16.51
Crude protein	12.18	13.02	12.63
Ether extract	2.87	3.30	3.39
Antinutritional factors			
Glycinin, mg/g	19.03	8.87	<2.00
β-conglycinin, mg/g	18.87	9.18	<2.00
Trypsin inhibitor, mg/g	<0.40	<0.40	<0.40
Agglutinin, mg/g	<0.20	<0.20	<0.20

STTD = standardized total tract digestible. SID, standardized ileal digestibility. ^1^ CON, the control group was fed a corn–soybean meal basal diet; CSM, the corn–soybean mixed meal group was fed with mixed meal to partially replace soybean meal in the basal diet; CMM, the corn mixed meal group was fed with mixed meal as a complete replacement of soybean meal in the basal diet. ^2^ The premix provided the following per kg of diets: VA 4 500 IU, VD2 100 IU, VE 22.5 mg, VK 3.75 mg, VB_1_ 2.25 mg, VB_2_ 7.5 mg, nicotinic acid 30 mg, D-pantothenic acid 11.25 mg, folic acid 0.75 mg, VB_6_ 3 mg, VB_12_ 0.03 mg, biotin 0.08 mg, Fe (FeSO_4_•H_2_O) 112.5 mg, Cu (CuSO_4_•5H_2_O) 6 mg, Mn (MnSO_4_•H_2_O) 4.5 mg, Zn (ZnSO_4_•H_2_O) 60 mg, I (CaI_2_O_6_) 0.14 mg, Se (Na_2_SeO_3_) 0.3 mg. ^3^ Calculated based on the values in the NRC (2012) [14].

**Table 2:** Effect of mixed meal replacement of soybean meal on the body size and organ indexes of finishing pigs.

Item	Treatments			*p*-Value
	CON	CSM	CMM	
Live weight, kg	126.16 ± 1.13	125.65 ± 1.36	125.14 ± 1.31	0.85
Body length, cm	146.83 ± 1.83	143.83 ± 0.91	142.83 ± 1.45	0.16
Body height, cm	74.83 ± 1.68	74.50 ± 1.73	75.83 ± 0.87	0.81
Chest circumference, cm	121.00 ± 2.35	116.67 ± 0.95	115.33 ± 2.03	0.12
Abdomen circumference, cm	119.83 ± 0.87	122.50 ± 2.22	126.00 ± 1.93	0.08
Heart index, %	0.35 ± 0.01	0.37 ± 0.02	0.36 ± 0.01	0.88
Liver index, %	1.59 ± 0.08	1.56 ± 0.05	1.46 ± 0.06	0.33
Spleen index, %	0.21 ± 0.01	0.19 ± 0.02	0.20 ± 0.01	0.77
Lung index, %	0.79 ± 0.08	0.75 ± 0.09	0.84 ± 0.12	0.83
Kidney index, %	0.29 ± 0.01	0.27 ± 0.01	0.78 ± 0.48	0.37

Data are presented as mean ± SEM (*n* = 6). CON, the control group was fed a corn–soybean meal basal diet; CSM, the corn–soybean mixed meal group was fed with mixed meal to partially replace soybean meal in the basal diet; CMM, the corn mixed meal group was fed with mixed meal as a complete replacement of soybean meal in the basal diet.

**Table 3:** Effect of mixed meal replacement of soybean meal on the carcass traits of finishing pigs.

Item	Treatments			*p*-Value
	CON	CSM	CMM	
Carcass straight length, cm	113.33 ± 1.67	113.50 ± 1.75	112.17 ± 0.83	0.79
Carcass oblique length, cm	102.67 ± 1.63	101.58 ± 1.45	102.50 ± 0.99	0.84
Carcass weight, kg	94.65 ± 1.59	92.64 ± 1.63	93.17 ± 0.83	0.59
Carcass yield, %	74.54 ± 1.02	74.08 ± 0.96	74.77 ± 0.65	0.86
Leaf fat weight, kg	1.49 ± 0.14 ^b^	1.51 ± 0.17 ^ab^	2.00 ± 0.09 ^a^	0.03
Average backfat thickness, mm	23.65 ± 0.79	27.73 ± 1.41	24.96 ± 1.43	0.09
Loin–eye area, cm^2^	67.83 ± 4.99	68.40 ± 5.92	63.33 ± 4.32	0.74

Data are presented as mean ± SEM (*n* = 6). Within a row, means without a common superscript letter differ at *p* < 0.05. CON: the control group was fed a corn–soybean meal basal diet; CSM: the corn–soybean mixed meal group was fed with mixed meal to partially replace soybean meal in the basal diet; CMM: the corn mixed meal group was fed with mixed meal as a complete replacement of soybean meal in the basal diet.

**Table 4:** Effect of mixed meal replacement of soybean meal on the meat quality of finishing pigs.

Item	Treatments			*p*-Value
	CON	CSM	CMM	
pH_45min_	6.43 ± 0.08	6.56 ± 0.15	6.39 ± 0.11	0.54
pH_24h_	5.53 ± 0.04	5.52 ± 0.03	5.52 ± 0.03	0.99
pH_48h_	5.60 ± 0.03	5.58 ± 0.02	5.57 ± 0.03	0.74
L*_45min_	16.86 ± 0.39	17.62 ± 0.87	16.76 ± 0.32	0.56
a*_45min_	6.69 ± 0.29	7.48 ± 0.53	6.46 ± 0.31	0.17
b*_45min_	61.77 ± 1.10	64.25 ± 0.86	61.70 ± 1.23	0.18
L*_24h_	16.83 ± 0.53	16.05 ± 0.58	16.72 ± 0.36	0.51
a*_24h_	8.69 ± 0.33	9.28 ± 0.17	8.61 ± 0.35	0.23
b*_24h_	62.26 ± 0.84	64.13 ± 1.10	61.54 ± 0.90	0.18
L*_48h_	18.60 ± 2.12	15.81 ± 0.43	16.84 ± 0.20	0.34
a*_48h_	9.20 ± 0.54	9.62 ± 0.44	8.55 ± 0.43	0.33
b*_48h_	16.86 ± 0.39	17.62 ± 0.87	16.76 ± 0.32	0.56
Drip loss_24h,_ %	2.45 ± 0.27	1.92 ± 0.33	2.33 ± 0.10	0.32
Drip loss_48h_, %	2.91 ± 0.21	3.76 ± 0.63	3.06 ± 0.18	0.31
Shear force, N	54.83 ± 3.61	53.13 ± 4.63	49.78 ± 5.14	0.73
Marbling scores	3.59 ± 0.29	3.69 ± 0.35	3.48 ± 0.22	0.88

Data are presented as mean ± SEM (*n* = 6). L*, brightness; a*, redness; b*, yellowness. CON: the control group was fed a corn–soybean meal basal diet; CSM: the corn–soybean mixed meal group was fed with mixed meal to partially replace soybean meal in the basal diet; CMM: the corn mixed meal group was fed with mixed meal as a complete replacement of soybean meal in the basal diet.

**Table 5:** Effect of mixed meal replacement of soybean meal on the longissimus lumborum fatty acid composition of finishing pigs (mg FA/100 g of muscle).

Item	Treatments			*p*-Value
	CON	CSM	CMM	
TFA	4716.72 ± 1345.29	3953.28 ± 678.62	3514.16 ± 659.01	0.67
SFA	2020.34 ± 590.67	1637.54 ± 303.22	1272.22 ± 205.30	0.44
C10:0	5.42 ± 1.56	4.76 ± 0.57	5.51 ± 0.69	0.86
C12:0	4.38 ± 1.32	4.35 ± 0.67	4.58 ± 0.72	0.98
C14:0	64.23 ± 18.40	59.11 ± 9.67	57.82 ± 10.61	0.94
C16:0	1224.87 ± 357.07	973.29 ± 189.84	587.33 ± 102.18	0.20
C17:0	11.40 ± 3.04	8.93 ± 1.88	8.59 ± 2.09	0.67
C18:0	697.55 ± 205.99	576.48 ± 105.35	597.33 ± 101.15	0.82
C20:0	10.80 ± 3.08	9.57 ± 1.49	10.11 ± 1.96	0.93
TUFA	2698.17 ± 754.71	2315.20 ± 384.16	2241.94 ± 460.71	0.83
MUFA	2045.12 ± 601.67	1689.16 ± 233.00	1615.64 ± 309.28	0.74
C16:1 (cis-9)	110.26 ± 32.02	104.14 ± 11.64	87.66 ± 14.03	0.74
C18:1 (trans-9)	3.66 ± 1.26	4.35 ± 0.48	4.60 ± 0.96	0.78
C18:1 (cis-9)	1890.90 ± 556.76	1547.68 ± 216.64	1492.28 ± 287.66	0.73
C20:1 (cis-11)	40.06 ± 12.53	32.99 ± 6.14	31.1 ± 7.09	0.77
PUFA	653.04 ± 155.63	626.04 ± 159.64	626.3 ± 156.58	0.99
C18:2 (all-cis-9,12)	553.40 ± 134.88	527.89 ± 138.99	526.39 ± 135.18	0.99
C18:3 (all-cis-9,12,15)	24.65 ± 5.97	27.42 ± 8.57	29.63 ± 8.41	0.90
C20:2 (all-cis-11,14)	26.51 ± 6.86	24.35 ± 7.26	23.5 ± 6.63	0.95
C20:3 (all-cis-8,11,14)	7.15 ± 1.28	6.44 ± 1.11	6.88 ± 1.26	0.92
C20:4 (all-cis-5,8,11,14)	33.18 ± 4.48	32.26 ± 2.32	31.85 ± 2.76	0.96
C20:3 (all-cis-11,14,17)	4.11 ± 1.51	5.06 ± 1.63	5.35 ± 1.63	0.85
C22:6 (all-cis-4,7,10,13,16,19)	1.72 ± 0.78	1.88 ± 0.63	1.65 ± 0.79	0.98

Data are presented as mean ± SEM (*n* = 6). CON: the control group was fed a corn–soybean meal basal diet; CSM: the corn–soybean mixed meal group was fed with mixed meal to partially replace soybean meal in the basal diet; CMM: the corn mixed meal group was fed with mixed meal as a complete replacement of soybean meal in the basal diet. Composition of fatty acids in LT is expressed in mg FA/100 g of fresh meat. TFA, total fatty acid; SFA, saturated fatty acid; TUFA, unsaturated fatty acids; MUFA, monounsaturated fatty acids; PUFA, polyunsaturated fatty acids.

**Table 6:** Effect of mixed meal replacement of soybean meal on the antioxidant capacity of longissimus thoracis in finishing pigs.

Item	Treatments			*p*-Value
	CON	CSM	CMM	
MDA, mmol/g port	0.21 ± 0.01	0.27 ± 0.04	0.20 ± 0.04	0.34
CAT, U/mg port	0.61 ± 0.05	0.40 ± 0.08	0.61 ± 0.10	0.11
T-SOD, U/mg port	50.22 ± 1.47	49.62 ± 1.03	48.19 ± 2.11	0.66
T-AOC, mmol/g port	57.44 ± 4.32	55.28 ± 5.00	50.29 ± 2.53	0.46

Data are presented as mean ± SEM (*n* = 6). CON, the control group was fed a corn–soybean meal basal diet; CSM, the corn–soybean mixed meal group was fed with mixed meal to partially replace soybean meal in the basal diet; CMM, the corn mixed meal group was fed with mixed meal as a complete replacement of soybean meal in the basal diet. MDA, malondialdehyde; T-AOC, total antioxidant capacity; T-SOD, total superoxide dismutase; CAT, catalase.

## Data Availability

The original contributions presented in this study are included in this article/Supplementary Materials; further inquiries can be directed to the corresponding author.

## Supplementary Materials

The following supporting information can be downloaded at: https://www.mdpi.com/article/10.3390/ani15111611/s1. Figure S1: Fatty acid profiles in feed determined using gas chromatography. Table S1: Fatty acid composition of diets. Table S2: The nutrient components of rapeseed meal, cottonseed meal, and sunflower seed meal.
