# Supplementary material for: Effects of Soybean Meal Substitution in Finishing Pig Diet on Carcass Traits, Meat Quality, and Muscle Antioxidant Capacity
Source: Animals (Basel). 2025 May 30;15(11):1611. doi: 10.3390/ani15111611 (PMC12153700; doi:10.3390/ani15111611)
Supplement: Supplementary file 1 [file animals-15-01611-s001.zip › animals-3604700-supplementary.pdf]

Supplementary Materials

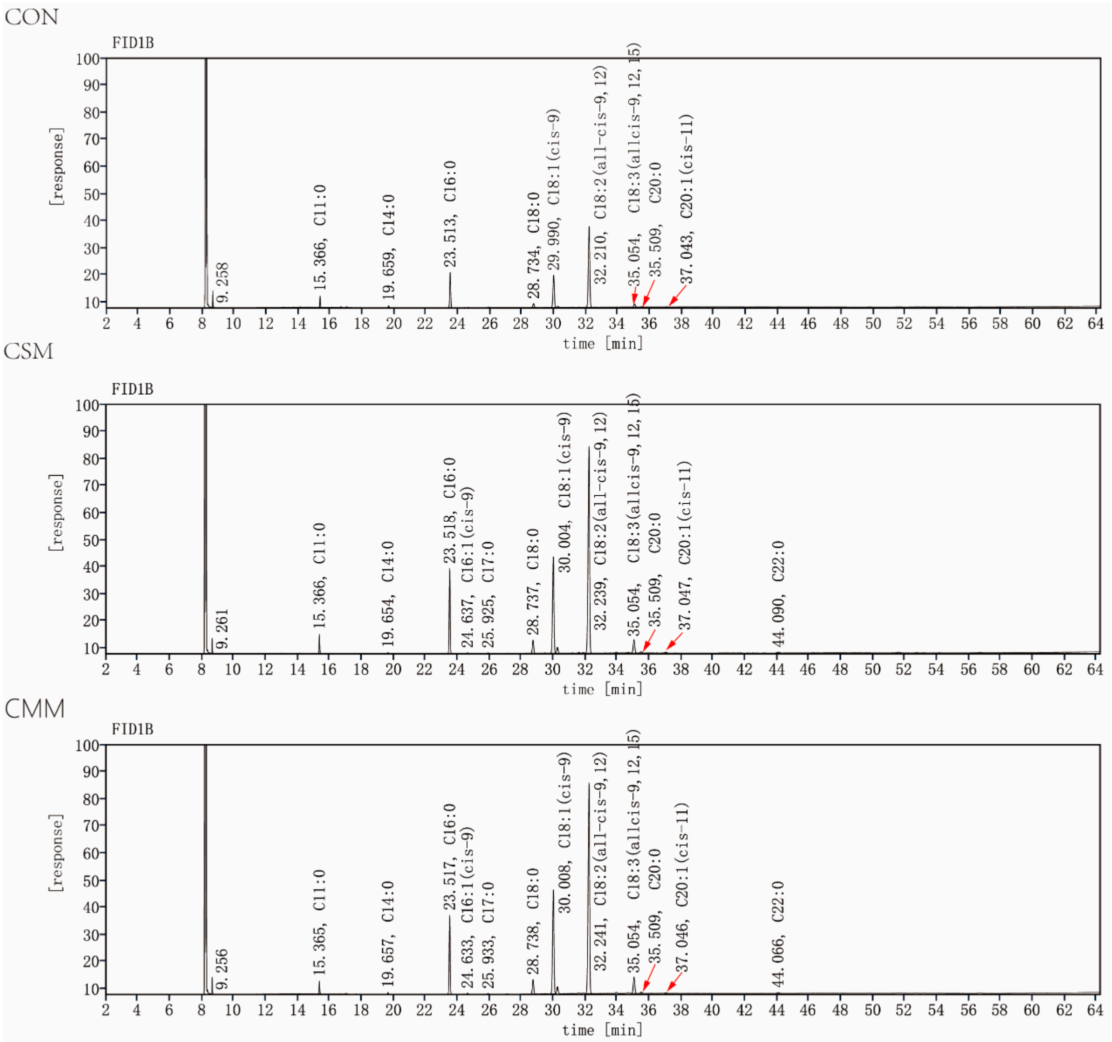

Figure S1. Fatty acid profiles in feed determined using gas chromatography. CON: the control group was fed corn-soybean meal basal diet; CSM: the corn-soybean meal mixed meal group was fed with mixed meal to partially replace soybean meal in the basal diet; CMM: the corn mixed meal group was fed with mixed meal as a complete replacement of soybean meal in the basal diet. Labelled as Retention time, fatty acids.

Table S1. Fatty acid composition of diets

Item	Feed		
	CON	CSM	CMM
C11:0	62.80	63.63	62.48
C14:0	12.58	6.38	10.53
C16:0	303.95	435.37	579.81
C16:1(cis-9)	/	5.50	7.94
C17:0	/	4.61	5.58
C18:0	41.23	85.07	136.07
C18:1(cis-9)	374.08	671.04	1023.63
C18:2(all-cis-9,12)	1021.79	1577.94	2274.63
C18:3(all-cis-9,12,15)	42.46	106.67	186.42
C20:0	6.68	10.96	15.72
C20:1(cis-11)	5.15	6.39	10.74
C22:0	/	7.81	14.15

CON: the control group was fed corn-soybean meal basal diet; CSM: the corn-soybean meal mixed meal group was fed with mixed meal to partially replace soybean meal in the basal diet; CMM: the corn mixed meal group was fed with mixed meal as a complete replacement of soybean meal in the basal diet. Composition of fatty acids in LT is expressed in mg FA/100 g of feed. / Indicates not detected in feed

Table S2. The nutrient components of rapeseed meal, cottonseed meal, and sunflower seed meal.

Item	Crude Protein %	Ether extract %	Crude Fiber %	Ca %	STTD P %
Rapeseed meal	38.60	1.40	11.80	0.65	0.25
Cottonseed meal	47.00	0.50	10.20	0.25	0.28
Sunflower seed meal	36.50	1.00	10.50	0.27	0.29

STTD = standardized total tract digestible.
